# Comparative efficacy and safety of oral pharmacological and complementary interventions for premenstrual syndrome: a protocol for a systematic review and a network meta-analysis

**DOI:** 10.1186/s13643-026-03255-1

**Published:** 2026-07-24

**Authors:** Bojana Degen, Cosima Locher, Helen Koechlin, Jens Gaab, Antje Frey Nascimento

**Affiliations:** 1 Division of Clinical Psychology and Psychotherapy, Faculty of Psychology, University of Basel, Missionsstrasse 62, Basel, 4055 Switzerland; 2 Department of Consultation-Liaison Psychiatry and Psychosomatic Medicine, University Hospital Zurich, University of Zurich, Zurich, Switzerland; 3 Clinical Psychology and Psychosomatics, Faculty of Psychology, University of Basel, Basel, Switzerland; 4 Department of Psychosomatic and Psychiatry, University Children’s Hospital Zurich, University of Zurich, Zurich, Switzerland; 5 Division of Child and Adolescent Health Psychology, Department of Psychology, University of Zurich, Zurich, Switzerland; 6 Children’s Research Centre University Children’s Hospital Zurich, University of Zurich, Zurich, Switzerland

**Keywords:** Premenstrual syndrome, Interventions, Network meta-analysis, Women’s health, Gynecological and reproductive medicine

## Abstract

**Background:**

Premenstrual syndrome (PMS) affects around 48% of women of reproductive age worldwide, interfering with psychological and physical well-being as well as functioning. A multitude of interventions exists for PMS, including oral pharmacological interventions, such as antidepressants, combined oral contraceptives, or alternative and complementary interventions. While the use of pharmacological interventions for PMS is linked to multiple side effects, evidence is limited for complementary interventions. Further, placebo effects are high in PMS intervention studies, affecting the estimation of the true treatment effect. An analysis of the relative efficacy between different treatment options is currently lacking, yet urgently needed for clinical decision-making.

**Methods:**

We plan a network meta-analysis to compare the relative efficacy and safety of oral pharmacological and non-pharmacological interventions for PMS, including all available randomized controlled trials, such as comparator or placebo-controlled trials. Primary outcomes will be symptom intensity and interference; secondary outcomes will be psychological and somatic symptom intensity. Safety will be assessed by the proportion of patients reporting treatment-related adverse events, unwanted events, and by dropouts due to side effects. Published trials will be retrieved from online electronic databases. At least two independent reviewers will screen, select references, and extract data independently. Risk of Bias will be assessed using the Cochrane risk of bias assessment 2 (RoB2) tool. We will conduct a random-effect network meta-analysis within a frequentist framework for each outcome to synthesize all available evidence and compare and rank available oral treatments.

**Discussion:**

Several interventions are available for PMS, a disabling condition that affects a large proportion of women worldwide. To our knowledge, this will be the first network meta-analysis to synthesize the existing evidence of oral PMS interventions systematically. Comparing the efficacy and safety of all available interventions is necessary to inform clinicians, patients and clinical guidelines to elect the best treatment regimen.

**Systematic review registration:**

PROSPERO CRD42020214716.

**Supplementary Information:**

The online version contains supplementary material available at https://doi.org/10.1186/s13643-026-03255-1.

## Background

Premenstrual syndrome (PMS) is defined by cyclically appearing psychological and/or physical symptoms that occur in the second half of the menstrual cycle (i.e., the luteal phase) and vanish at menstruation onset or briefly after, leading to considerable interference in daily life [1, 2]. While psychological symptoms entail irritability, sadness, and mood swings, somatic symptoms include breast tenderness, bloating, and joint pain [3]. Around 48% of women of reproductive age suffer worldwide from PMS, with a broad variability in prevalence between countries, ranging from 12% for France to 98% for Iran [4]. The considerable difference in prevalence rates across countries could be due to cultural variations of PMS appraisal, differences in age, physical activity, or nutrition of the respective study populations, and the use of different PMS assessment tools [4, 5].

Premenstrual dysphoric disorder (PMDD), a severe form of PMS that includes different affective symptoms such as irritability, mood swings, and depressive feelings, is classified as a mental disorder in the Diagnostic and Statistical Manual of Mental Disorders of the American Psychiatric Association (DSM-5-TR; [6]). Prevalence rates for PMDD range between 3.2% for confirmed and 7.7% for provisional diagnosis [7].

Women with PMS and PMDD report significant psychological and physical strain with an increased risk of suicidality and symptoms that are comparably disabling to those of major depression [8]. Consequently, affected women experience a negative impact on their social life and relationships, as well as on their work or school performance, leading to increased work absenteeism and, thus, to economic consequences on a social level [9, 10].

For several decades, along with a steady transition of the concept of PMS and PMDD, it has been a huge challenge to find effective interventions for PMS and PMDD [8, 11]. In contrast, today, a myriad of treatment options is obtainable [1, 12]. However, precise mechanisms of action of different PMS and PMDD interventions are still unclear since PMS and PMDD contain a complex etiological pathophysiology with the involvement of multiple neuro-hormonal systems and pathways [10, 13, 14]. The most prevailing approach to treat PMS and PMDD is oral pharmacological agents, comprising first-line interventions including selective serotonin reuptake inhibitors (SSRI), hormonal agents such as combined oral contraceptives (COC), and Danazol (a testosterone derivative), as well as Gonadotropin-releasing hormone analogues for severe cases [15]. Previous meta-analyses show small to medium effect sizes for recommended PMS and PMDD interventions compared to placebo (e.g., SSRIs: standardized mean difference [SMD] = 0.36; COC: SMD = 0.41; [15–17]). Notably, a considerable array of side effects is reported for first-line interventions, including nausea, tremor, and dizziness, for instance, for SSRIs, and gastrointestinal symptoms, weight gain, and depression for Danazol [2, 10]. As a result, women often discontinue intake when treated with SSRIs for PMS and PMDD due to treatment-emerged adverse events, leading to diminished adherence [17].

A further treatment modality comprises oral alternative and complementary interventions and supplements, for instance, Vitex agnus castus (i.e., herbal medicine), vitamin supplements, and magnesium [18–21]. While Vitex agnus castus was shown to be efficacious compared to placebo [19, 21], inconsistent evidence is reported for other frequently prescribed or self-administered complementary and alternative oral treatments, such as vitamin supplements or magnesium [18, 22, 23].

Notably, and especially when compared to pharmacological interventions, placebo effects are considerably high in PMS and PMDD, ranging from 36 to 43% [15, 24, 25]. Given that evidence-based interventions have to be significantly more efficacious than control groups (i.e., usually placebos), and considering the detrimental side effects of first-line PMS treatments, a careful evaluation of the best treatment approach is deemed necessary [1, 17, 26]. Besides oral interventions, lifestyle changes such as more exercise, symptom management by means of symptom diary usage, and dietary changes are also recommended to improve PMS and PMDD [1].

Recent guidelines endorse the implementation of shared decision-making in clinical practice, comprising the consideration of patients’ preferences and objectives in the treatment of PMS, requiring a sound elucidation of available treatment alternatives with their risks and benefits [1]. Furthermore, available single-intervention meta-analyses (such as existing Cochrane Reviews on SSRI`s or COC) only compare intervention with placebo and ignore indirect comparisons across interventions. While this helps us understand whether a single intervention works better than a placebo, it does not allow us to compare how treatments score against each other. Therefore, it does not inform clinicians which treatment is more effective or safer relative to the others. Finally, single-intervention meta-analyses often produce fragmented knowledge, which can lead to conflicting conclusions or incomplete synthesis.

Here, the lack of a comprehensive understanding regarding the relative efficacy and safety of available oral interventions for PMS and PMDD forms a fundamental barrier in the election of the best treatment regimen for affected women in the clinical setting and impedes solid information of patients. As network meta-analyses synthesize all available evidence into a single, connected framework, they enable a comprehensive ranking of treatments by efficacy and safety, and help improve decision-making when head-to-head trials are missing. Therefore, we will conduct a network meta-analysis to compare the relative efficacy and safety of available oral pharmacological interventions for PMS and PMDD, as this uniquely allows us to combine direct and indirect evidence.

### Objectives

We will synthesize available data from randomized controlled trials (RCTs), examining oral interventions for PMS and PMDD. Our primary objective is to compare the efficacy (i.e., reduction of symptom intensity and interference) and assess intervention safety (i.e., side effects and study discontinuation due to side effects) of all available oral interventions for PMS and PMDD with each other and/or oral placebo control groups. We will focus on post-treatment effects and relative efficacy for interventions and control conditions.

## Methods and analysis

### Protocol and registration

This protocol is written following the PRISMA-P guidelines (see Additional file 1; [27]), and is registered on PROSPERO under the registration number CRD42020214716 (available from http://www.crd.york.ac.uk/PROSPERO).

### Eligibility criteria for considering studies for review

#### Types of studies

All randomized controlled trials that examine available oral interventions (e.g., pharmacological, complementary, or supplements) for women suffering from PMS or PMDD and report at least one outcome as defined below will be included. The diagnostic process in included studies must rely on standardized prospective diagnostic screening tools for premenstrual disorders (e.g., Daily Record of Severity of Problems: DRSP). Trials need to make head-to-head comparisons of two different oral interventions (i.e., comparator trial) or compare an intervention with an oral placebo control (i.e., placebo-controlled trials) to be included. Furthermore, cross-over trials will be included if the results of the first cross-over sequence are reported separately. Randomized single-control studies (e.g., comparison of different dosages of one intervention) will be included only if oral placebo control groups exist as defined above. Different dosages of the same intervention will be merged.

Conference papers will not be included in our review. Also, RCTs in which participants with PMS or PMDD additionally suffered from comorbid psychiatric disorders (e.g., depression, bipolar disorder, anxiety disorders), as well as menstrual migraine, cyclical mastalgia, or premenstrual exacerbation of other underlying disorders, will be excluded. We will also exclude case–control studies, post hoc and secondary analyses.

#### Types of participants

We will include RCTs with women of reproductive age who fulfill diagnostic criteria for premenstrual disorders defined by the consensus of the International Society for Premenstrual Disorders (ISPMD; [5, 28, 29]) comparable to the PMS definition as described in the guideline of the Royal College of Obstetricians and Gynecologists (RCOG; [15]). Furthermore, RCTs with women fulfilling PMDD diagnostic criteria in agreement with the DSM classification will be included. Conformity of diagnostic criteria as reported in included RCTs with the definition of PMS according to RCOG, ISPMD, or DSM-5-TR criteria for PMDD will be determined by two authors independently and must rely on standardized prospective diagnostic screening tools for premenstrual disorders (e.g., DRSP; [2, 5, 15, 28]). For our network meta-analyses, we rely on the definition of PMS as the presence of any clinically significant symptom(s), such as psychological (i.e., affective or behavioral symptoms, such as irritability, depression, feeling anxious) or physical symptom (such as breast tenderness, general pain) in the luteal phase of the menstrual cycle, causing substantial distress and functional impairment [5]. PMDD is defined according to the DSM-5-TR or earlier editions [6], entailing at least five symptoms out of two symptom clusters (i.e., 1st cluster: affect lability, anger, conflicts with others; 2nd cluster: loss of interest, lack of concentration, lethargy) causing as well substantial distress and functional impairment.

#### Types of interventions

We will include all available oral pharmacological and complementary interventions for PMS and PMDD. Oral pharmacological interventions targeting PMS or PMDD symptoms, as well as oral complementary interventions, have to be administered in any form orally (e.g., pills, dragées [i.e., sugar-coated tablet], suspension, or powder). In addition, we will only include patients treated for at least one average menstrual cycle (i.e., 4 weeks; [5, 13, 30]), regardless of the setting (in- or outpatient).

#### Type of comparators

Each RCT must directly compare at least two active oral interventions with each other (comparator trials) or with an oral placebo group (placebo-controlled trials).

#### Efficacy outcome measures

Our choice of outcomes is based on recommendations for clinical trials studying PMS and PMDD [5, 29], and on reported outcomes in existing meta-analyses and reviews investigating distinct oral treatments for PMS and PMDD [17, 19, 21, 22]. All outcomes must rely on a prospective daily assessment of patient self-reported PMS complaints (e.g., DRSP), as retrospective assessment of PMS/PMDD (e.g., Premenstrual Symptoms Screening Tool) shows less intra-reliability compared to prospective continuous assessment [15, 29, 31]. We will conduct separate network meta-analyses for each outcome (see following outcomes).

#### Primary outcomes

Difference scores of overall PMS and PMDD (i) symptom intensity and (ii) interference from baseline to post-intervention are considered as primary outcomes, if reported, measured with a prospective daily assessment (e.g., DRSP, MDQ, DSR), independent of type of symptoms, considering the overall score, and determined by two researchers independently, to ensure consistency of assessment tools with diagnostic criteria. Standardized mean differences will be used to compare effects independent of scales. Whenever possible, outcomes of heterogeneous scales will be transformed into a common, comparable scale by harmonization. A methodological and theoretical evaluation will be used in this case to assess whether pooling data is appropriate for the respective questionnaire and diary data.

#### Secondary outcomes

Our secondary outcomes are difference scores from baseline to post-intervention of (i) overall psychological symptom intensity and (ii) overall somatic symptom intensity in PMS and PMDD, measured as a subscale of overall symptom intensity. However, if these outcomes are not reported as overall scores but as individual scores for multiple symptoms (i.e., at least two psychological or somatic symptoms), overall scores of psychological symptom intensity and somatic symptom intensity will be calculated. If only one individual score for either psychological or somatic symptom intensity is reported, we will treat the reported symptom intensity for the respective single symptom as an overall score. Symptoms will be classified as psychological or somatic PMS symptoms according to the classification of Ditzen et al. [32].

#### Safety outcome measures

Safety will be assessed by reported treatment-related adverse events, such as serious adverse events (SAEs), treatment-emergent adverse events (TEAEs), and unwanted events (UEs). In this regard, we will extract (1) the mean number of SAEs, TEAEs, or UEs per patient across all reported outcomes (i.e., for the post-intervention time point, as defined below if the outcome is reported); (2) the proportion of patients reporting SAEs, TEAEs, and UEs; (3) study discontinuation due to side effects.

#### Search strategy and study selection

The search strategy focused on the search of online electronic databases (PsycINFO, Psyndex, MEDLINE, PubMed, Embase, Scopus, Web of Science, EBSCO Health/CINAHL, Cochrane) searching each from inception until 13 June 2023 for publications of studies that investigated an oral pharmacological or complementary intervention for women suffering from PMS and PMDD without restrictions to language. The detailed search strategy is described in the Additional file 2. Results will be exported into Covidence, a web-based software recommended by the Cochrane Network for the conduct of systematic reviews (https://www.covidence.org). Two reviewers will independently screen titles and abstracts in the selection process of each paper. Conflicts will be resolved through the consensus of one of the two primary investigators (BD and AFN). If no consensus can be reached, conflicts will be discussed in the study team and resolved by a third reviewer (CL). Studies referring to clinical trial registrations, found in the titles and abstracts screening, are searched manually and added whenever accessible. The full text of all articles included by reference and abstract screening will be retrieved and will be screened independently by two reviewers using the eligibility criteria mentioned above. If information on these domains in the article is missing, we will attempt to contact the trial authors to obtain further information.

In case the data extraction takes longer than expected, we will consider updating the search described above.

## Data extraction (selection and coding)

Two independent reviewers will extract all relevant data from the trial reports. Disagreements will be discussed by the two reviewers or, if necessary, through the consultation of a third reviewer. Data will be extracted into a structured form. Extracted data will include study design (e.g., parallel or cross-over design), study characteristics (e.g., lead author, publication year, journal, sponsor, region, setting), participant characteristics (e.g., diagnostic criteria, age, duration of symptoms, demographic data), intervention details (e.g., type of intervention, treatment regimen, drug dose, a dosing schedule like continuous or intermittent, length of intervention, co-intervention), placebo group details (e.g., placebo lead-in yes/no, placebo administration form, dose, and dosing schedule, length of placebo), analysis details (e.g., intent-to-treat, secondary or sub-analysis), and outcome measures (e.g., primary, secondary, and safety outcomes). The preliminary data extraction form will be piloted independently by two reviewers on 10 randomly selected studies. This process will enable the modification of the structured pilot data chart based on experiences of extracting data from each article before conducting final data extraction.

### Outcome measurements

To calculate relative treatment effects, we will extract means and standard deviations (SDs) for continuous outcomes (i.e., primary and secondary outcomes). For categorical outcomes (i.e., primary, secondary, or safety outcomes), the number of events and the total number of occurrences will be extracted. Data extraction will be done using a structured Excel spreadsheet in a wide format (i.e., single study arm per row) for continuous and categorical outcomes.

### Missing outcome data

If SDs are not provided for the continuous outcomes, we will calculate them from standard errors (SEs), confidence intervals, and other measures, or otherwise impute the mean of SDs from studies using the same outcome measure [33].

In this regard, sensitivity analyses will be conducted, including all studies, those with imputed SDs vs. only studies that report SDs. If a significant difference exists, studies with missing SDs will be excluded. If the number of patients included in the analysis is missing, we will use the number of overall randomized patients in each study arm. If continuous outcome data are not reported, we will extract the number, respectively, the percentage of patients that fulfilled the response criterion as defined by the respective study authors (e.g., overall symptom reduction), and the number of patients that did not fulfill the respective criterion [34].

### Length of trial

Furthermore, we defined the following time points of measurements: at least 4 weeks (i.e., one average menstrual cycle) up to 12 weeks (i.e., three menstrual cycles) after randomization as postintervention values, whereas the values at the time of randomization are considered baseline. If defined post-intervention data are unavailable in this timeframe, we will assess the data for post-intervention at the end of each intervention, closest to the three menstrual cycles of intervention.

## Risk of bias in individual studies

We will assess the risk of bias for selected outcomes of each included study using the Cochrane Risk of Bias Assessment 2 (RoB2) tool [35]. Two independent raters will assess the risk of bias for each included study. Disagreements will be resolved through discussion or consultation with a third reviewer. Risk of bias will be assessed for primary and safety outcomes and will be rated across the following domains: randomization process, deviations from intended interventions, missing outcome data, outcome reporting, and reported results.

## Statistical synthesis of study data

### Description of the network

For each included trial, we will present descriptive statistics, including the types of comparisons and study characteristics described above. We will present the results in a network diagram for each outcome. The size of the nodes will reflect the quantity of evidence for each treatment in a total number of patients. The width of each edge will be proportional to the number of studies showing direct treatment comparison, and the color of each edge will represent the RoB2 assessment.

### Pairwise meta-analyses

Mean differences of efficacy outcomes between treatment groups will be estimated for each study and pooled using a standardized mean difference (SMD). For safety outcomes, we will calculate odds ratios (ORs) with 95% confidence intervals.

### Network meta-analyses

To calculate network meta-analyses within a frequentist framework, the package netmeta [36, 37] for the open-source software environment RStudio, a recent version for Mac OS, will be used. A network will be created, including all available comparisons. We will start by splitting the interventions and plotting networks first. In the case of an unconnected network, interventions might be classified into larger groups (i.e., lumping approach) based on similar components. Random-effects pairwise SMD will be calculated based on direct comparisons for the continuous outcomes. Risk (i.e., probability of study discontinuation due to side effects) and risk rate ratios (i.e., the proportion of patients and mean number of SAEs, TEAEs, or UEs) will be calculated for categorical outcomes. In addition, indirect evidence will be estimated using the entire evidence network. Random effects account for dependencies between comparisons in the case of multi-arm trials, and we assume heterogeneity across included studies. Results from the network meta-analysis will be presented as a summary SMD with 95% confidence intervals (CIs) for all relevant pairs of treatments in a league table.

In the case of an unconnected network (e.g., two separate networks for pharmacological vs. complementary interventions), we plan to conduct and report results from each network analyzed separately.

### Certainty of evidence

The certainty of evidence in network estimates of the main outcomes will be assessed using the Grading of Recommendations Assessment, Development, and Evaluation (GRADE) ratings, applying the CINEMA framework [38, 39]. This assessment includes study limitations (within-study bias), imprecision, incoherence, heterogeneity, indirectness, and publication bias (across-studies bias).

### Transitivity assumption and assessment of consistency

The transitivity assumption states that direct evidence, e.g., from AC and BC studies, can be combined to indirectly learn about the comparison AB. We will address the assumption of transitivity by considering three of its equivalent expressions: (1) comparability of studies in terms of distribution of effect modifiers; (2) statistical agreement of direct and indirect treatment effects (via an assessment for inconsistency); and (3) the principle of jointly randomizability, i.e., whether participants in the whole network could be randomized to any of the included treatments [40].

To statistically evaluate consistency, which describes the agreement between direct and indirect evidence, we will use local (i.e., the separation of direct from indirect evidence) and global approaches (i.e., design-by-treatment interaction test) [41, 42].

### Assessment of heterogeneity

Heterogeneity will be examined by calculating the Q statistic, the τ2, and the *I*^2^, a transformation of Q that indicates the proportion of observed variance that can be attributed to heterogeneity rather than sampling error [43, 44]. However, we will prefer the Q statistic and the τ2 over the *I*^2^, as the *I*^2^ is sensitive to sample size [45]. To further understand the clinical relevance of heterogeneity in our networks, we will calculate prediction intervals to estimate the true effects that can be expected in future settings.

### Subgroup analyses and sensitivity analyses

Subgroup analyses are planned to explore if treatment regimen, drug dose (high-dose vs. low-dose), dosing schedule (continuous vs. intermittent), length of intervention (intervention length ≤ 3 months vs. intervention length > 3 months), co-intervention, sociodemographic characteristics, or country of origin/region potentially have an effect on the primary outcomes. Sensitivity analyses will include (a) imputed vs. reported SDs, (b) studies with un- vs. adjusted means, (c) high risk of bias vs low risk of bias. Sources of heterogeneity or inconsistency will be explored by excluding outliers and conducting additional subgroup analyses if substantial heterogeneity or inconsistency is observed.

### Selection bias

Comparison-adjusted and contour-enhanced funnel plots will be applied to investigate whether there is a significant difference in results from imprecise vs more precise trials [46, 47]. We will further perform a meta-regression, using a measure of study precision as a covariate, allowing us to explore potential issues with small study effects [48].

## Discussion

Premenstrual disorders are highly prevalent among women of reproductive age, with a wide range of different treatment options and modalities available. Different meta-analyses have been conducted to investigate the efficacy of single oral interventions for PMS and PMDD, such as SSRIs [17, 49], combined oral contraceptives [16, 50], and oral alternative and complementary interventions [18, 19, 21, 22], adding to the knowledge about efficacy and safety in the treatment of PMS and PMDD. Remarkably, significant placebo effects [16, 19, 23], as well as considerable safety issues, are reported across meta-analyses, with the most effective interventions encompassing severe side effects and, thus, increasing non-adherence (e.g., [17]). Since, for PMS and PMDD, a large array of interventions is available, meta-analyses can only show a small excerpt of the whole picture concerning intervention efficacy and safety. Furthermore, no evidence exists of how efficacy and safety differ between interventions. Here, a network meta-analytic approach would enable the comparison of the efficacy and safety of multiple interventions for PMS and PMDD and is highly needed, given the many available PMS and PMDD interventions [17].

Thus, we will synthesize all available evidence of the efficacy and safety of available oral PMS and PMDD interventions into a network to provide an overview of the relative efficacy and safety of available interventions, enabling direct and indirect comparisons as well as ranking of the efficacy and safety of different oral interventions. To our knowledge, this will be the first network meta-analysis of oral interventions of PMS and PMDD. Our approach will enable us to attain the most precise estimates of the differences between interventions and, therefore, offer a more comprehensive understanding of PMS and PMDD oral interventions in terms of their risk–benefit ratio and suitability for different PMS symptom clusters (i.e., psychological and somatic symptoms). The results of our network meta-analyses aim to provide soil for clinicians to decide informedly, together with patients suffering from PMS and PMDD, for the most suitable and acceptable intervention, thereby improving pharmacological PMS and PMDD management. Hence, the efficacy and safety rankings of interventions can help to inform current guidelines of PMS/PMDD treatment. Further, they may be utilized for clinical decision-making and shared decision-making by tailoring a fitting treatment regimen, considering as well individual patient preferences (e.g., intervention modality and length) and characteristics (e.g., medical conditions and more sensitivity to side effects). With this aim, our data may serve to plot individual cost–benefit profiles for each intervention included in the network.

### Limitations

Even though this NMA is designed to synthesize the best available evidence, its conclusions will rely on the quality and structure of the network. Risk of bias, network unconnectivity, and heterogeneity may all potentially limit the certainty and the reliability of comparative estimates and will be evaluated and reported in the publication together with the results. Furthermore, exclusion of the studies with comorbid psychiatric disorders may potentially limit the generalizability of the study, given the frequent overlap of PMS and PMDD with psychiatric disorders. Finally, our search strategy did not encompass grey literature (e.g., ClinicalTrials.gov), which can potentially introduce publication bias.

## Supplementary Information

Additional file 1. PRISMA‐P 2015 Checklist.

Additional file 2: Search strategy.

## Data Availability

The datasets analyzed during the current study are available from the corresponding author on reasonable request.

## Abbreviations

CI: Confidence interval
COC: Combined oral contraceptives
PMS: Premenstrual syndrome
PMDD: Premenstrual dysphoric disorder
PRISMA-P: Preferred Reporting Items for Systematic Review and Meta-Analysis Protocols
PROSPERO: International Prospective Register of Systematic Reviews
RCT: Randomized-controlled trial
SSRI: Selective serotonin reuptake inhibitor
