# Supplementary material for: Comparative efficacy and safety of oral pharmacological and complementary interventions for premenstrual syndrome: a protocol for a systematic review and a network meta-analysis
Source: Syst Rev. 2026 Jul 24;15:276. doi: 10.1186/s13643-026-03255-1 (PMC13685308; doi:10.1186/s13643-026-03255-1)
Supplement: Supplementary file 2 [file 13643_2026_3255_MOESM2_ESM.docx]

**Additional File 2.**

**Comparative efficacy and safety of oral pharmacological and complementary interventions for premenstrual syndrome: Search strategies and hits**

| **Database Searched** | **Date Searched** | **Results Returned** |
| --- | --- | --- |
| **PsycINFO** | 13/06/2023 | 312 |
| **Psyndex** | 13/06/2023 | 9 |
| **MEDLINE** | 13/06/2023 | 849 |
| **PubMed** | 13/06/2023 | 804 |
| **Embase** | 13/06/2023 | 11 044 |
| **Scopus** | 13/06/2023 | 2 469 |
| **Web of Science** | 13/06/2023 | 1 419 |
| **EBSCO Health/CINAHL** | 13/06/2023 | 441 |
| **Cohrane** | 13/06/2023 | 852 |
| **Database Total** | **---** | **18 199** |

***PsycINFO*** *(Ovid, Advanced Search)*

(conducted 2023-06-13; 312 hits)

((premenstruum OR "premenstrual syndrome" OR "premenstrual symptom*" OR PMS OR "premenstrual dysphoric disorder*" OR PMDD OR "premenstrual disturbance*" OR "premenstrual disorder*" OR PMD OR "premenstrual complaint*"

OR "premenstrual ailment" OR "premenstrual burden" OR "premenstrual tension" OR PMT OR "premenstrual tension syndrome" OR PMTS OR "late luteal

phase dysphoric disorder" OR LLPDD OR "premenstrual dysphoria" OR "premenstrual distress" OR "premenstrual mood change*" OR "premenstrual depression" OR premenstrual).ti,ab,id

OR ("Premenstrual Syndrome" OR "Premenstrual Dysphoric Disorder" OR " Menstrual Disorders" OR "Menstrual Cycle").sh

OR ("Premenstrual Syndrome" or "Premenstrual Dysphoric Disorder").mh)

AND

(("controlled stud*" OR "controlled trial*" OR "clinical trial*" OR randomi?ed OR "randomi?ed controlled trial*" OR RCT OR blind OR "double blind").ti,ab,id

OR ("Clinical Trials" OR "Treatment Effectiveness Evaluation").sh

OR ("Double-Blind Method" or "Randomized Controlled Trial" or "Clinical Trial").mh)

AND

((phytopharmaceutical* OR "herbal medicine" OR "complementary medicine" OR "alternative medicine" OR "st. john's wort" OR "saint john's wort" OR "hypericum perforatum" OR "vitex agnus castus" OR

"black cohosh" OR "actaea racemosa" OR "neptune krill" OR valerian OR "valeriana officinalis" OR balm OR melissa OR saffron OR crocus OR "crocus sativus" OR "evening primrose oil" OR

pharmacotherapy OR pharmacotherapeutics OR "pharmacological treatment*" OR "non-hormonal treatment*" OR anxiolytic* OR tranquilizer* OR benzodiazepine OR buspirone OR alprazolam OR antidepressant* OR

"tricyclic antidepressant*" OR "serotonin reuptake inhibitor*" OR SRI OR "selective serotonin reuptake inhibitor*" OR SSRI OR clomipramine OR fluoxetine OR paroxetine OR sertraline OR

citalopram OR escitalopram OR fluvoxamine OR venlafaxine OR "L-tryptophan" OR "norepinephrine reuptake inhibitor*" OR NRI OR "noradrenaline reuptake inhibitor*" OR NERI OR

"adrenergic reuptake inhibitor*" OR ARI OR Lithium OR analgesic* OR paracetamol OR acetaminophen OR aspirin OR "acetylsalicylic acid" OR ASA OR "beta-blocker*" OR diuretics OR spironolactone OR

"nonsteroidal anti-inflammatory drug*" OR NSAID OR "mefenamic acid" OR ibuprofen OR "hormonal treatment*" OR "hormone therap*" OR progestins OR progesterone OR drospirenone OR norgestimate OR

levonorgestrel OR desogestrel OR norethindrone OR "cyproterone acetate" OR "oral contraceptive*" OR "combined oral contraceptive pill" OR COCP OR "gonadotropin-releasing hormone agonist" OR

"GnRH agonist*" OR "GnRH agonist analogs" OR progestogens OR norethisterone OR dydrogesterone OR "medroxyprogesterone acetate" OR "depo-provera" OR testosterone OR bromocriptine OR mifepristone

OR "RU-486" OR "cyproterone acetate" OR tibolone OR danazol OR gestrinone OR estradiol OR oestradiol OR "dietary supplement*" OR "vitamin B6" OR "vitamin D" OR calcium OR magnesium OR zinc).ti,ab,id

OR ("Medicinal Herbs and Plants" OR "Alternative Medicine" OR "Hypericum Perforatum" OR "Drug Therapy" OR "Tranquilizing Drugs" OR BENZODIAZEPINES OR BUSPIRONE OR ALPRAZOLAM OR "ANTIDEPRESSANT DRUGS"

OR "TRICYCLIC ANTIDEPRESSANT DRUGS" OR "Serotonin Reuptake Inhibitors" OR Chlorimipramine OR FLUOXETINE OR PAROXETINE OR SERTRALINE OR CITALOPRAM OR FLUVOXAMINE OR VENLAFAXINE OR Tryptophan OR

"Serotonin Norepinephrine Reuptake Inhibitors" OR LITHIUM OR "ANALGESIC DRUGS" OR Aspirin OR "Adrenergic Blocking Drugs" OR DIURETICS OR "Anti Inflammatory Drugs" OR "Hormone Therapy" OR "Progestational Hormones"

OR PROGESTERONE OR "Oral Contraceptives" OR TESTOSTERONE OR Bromocriptine OR ESTRADIOL OR "Dietary Supplements" OR Vitamins OR CALCIUM OR MAGNESIUM OR ZINC).sh

OR ("Herbal Medicine" OR "Complementary Therapies" OR Hypericum OR Vitex OR Cimicifuga OR Valerian OR Melissa OR Crocus OR "Oenothera biennis" OR "Drug Therapy" OR "Anti-Anxiety Agents" OR "Tranquilizing Agents"

OR Benzodiazepines OR Buspirone OR Alprazolam OR "Antidepressive Agents" OR "Antidepressive Agents, Tricyclic" OR Serotonin and "Noradrenaline Reuptake Inhibitors" OR "Serotonin Uptake Inhibitors"

OR Clomipramine OR Fluoxetine OR Paroxetine OR Sertraline OR Citalopram OR Fluvoxamine OR "Venlafaxine Hydrochloride" OR Tryptophan OR "Adrenergic Uptake Inhibitors" OR "Lithium" OR Analgesics OR Acetaminophen

OR Acetaminophen OR Aspirin OR "Adrenergic beta-3 Receptor Antagonists" OR Diuretics OR Spironolactone OR "Anti-Inflammatory Agents, Non-Steroidal" OR "Mefenamic Acid" OR Ibuprofen OR Progestins

OR Progesterone OR Levonorgestrel OR Desogestrel OR Norethindrone OR "Cyproterone Acetate" OR "Contraceptives, Oral" OR "Contraceptives, Oral, Combined" OR Progestins OR Norethindrone OR Dydrogesterone

OR "Medroxyprogesterone Acetate" OR Testosterone OR Bromocriptine OR Mifepristone OR "Cyproterone Acetate" OR Danazol OR Gestrinone OR Estradiol OR "Dietary Supplements" OR "Vitamin B 6" OR "Vitamin D" OR

Calcium OR Magnesium OR Zinc).mh)

***PSYNDEXplus Literature and Audiovisual Media*** *(Ovid, Advanced Search)*

(conducted 2023-06-13; 9 hits)

((premenstruum OR "premenstrual syndrome" OR "premenstrual symptom*" OR PMS OR "premenstrual dysphoric disorder*" OR PMDD OR "premenstrual disturbance*" OR "premenstrual disorder*" OR PMD

OR "premenstrual complaint*" OR "premenstrual ailment" OR "premenstrual burden" OR "premenstrual tension" OR PMT OR "premenstrual tension syndrome" OR PMTS OR "late luteal

phase dysphoric disorder" OR LLPDD OR "premenstrual dysphoria" OR "premenstrual distress" OR "premenstrual mood change*" OR "premenstrual depression" OR premenstrual).ti,ab,id

OR ("Premenstrual Syndrome" OR "Premenstrual Dysphoric Disorder" OR " Menstrual Disorders" OR "Menstrual Cycle").sh)

AND

(("controlled stud*" OR "controlled trial*" OR "clinical trial*" OR randomi?ed OR "randomi?ed controlled trial*" OR RCT OR blind OR "double blind").ti,ab,id

OR ("Clinical Trials" OR "Treatment Effectiveness Evaluation").sh)

AND

((phytopharmaceutical* OR "herbal medicine" OR "complementary medicine" OR "alternative medicine" OR "st. john's wort" OR "saint john's wort" OR "hypericum perforatum" OR "vitex agnus castus" OR

"black cohosh" OR "actaea racemosa" OR "neptune krill" OR valerian OR "valeriana officinalis" OR balm OR melissa OR saffron OR crocus OR "crocus sativus" OR "evening primrose oil" OR

pharmacotherapy OR pharmacotherapeutics OR "pharmacological treatment*" OR "non-hormonal treatment*" OR anxiolytic* OR tranquilizer* OR benzodiazepine OR buspirone OR alprazolam OR antidepressant* OR

"tricyclic antidepressant*" OR "serotonin reuptake inhibitor*" OR SRI OR "selective serotonin reuptake inhibitor*" OR SSRI OR clomipramine OR fluoxetine OR paroxetine OR sertraline OR

citalopram OR escitalopram OR fluvoxamine OR venlafaxine OR "L-tryptophan" OR "norepinephrine reuptake inhibitor*" OR NRI OR "noradrenaline reuptake inhibitor*" OR NERI OR

"adrenergic reuptake inhibitor*" OR ARI OR Lithium OR analgesic* OR paracetamol OR acetaminophen OR aspirin OR "acetylsalicylic acid" OR ASA OR "beta-blocker*" OR diuretics OR spironolactone OR

"nonsteroidal anti-inflammatory drug*" OR NSAID OR "mefenamic acid" OR ibuprofen OR "hormonal treatment*" OR "hormone therap*" OR progestins OR progesterone OR drospirenone OR norgestimate OR

levonorgestrel OR desogestrel OR norethindrone OR "cyproterone acetate" OR "oral contraceptive*" OR "combined oral contraceptive pill" OR COCP OR "gonadotropin-releasing hormone agonist" OR

"GnRH agonist*" OR "GnRH agonist analogs" OR progestogens OR norethisterone OR dydrogesterone OR "medroxyprogesterone acetate" OR "depo-provera" OR testosterone OR bromocriptine OR mifepristone

OR "RU-486" OR "cyproterone acetate" OR tibolone OR danazol OR gestrinone OR estradiol OR oestradiol OR "dietary supplement*" OR "vitamin B6" OR "vitamin D" OR calcium OR magnesium OR zinc).ti,ab,id

OR ("Medicinal Herbs and Plants" OR "Alternative Medicine" OR "Hypericum Perforatum" OR "Drug Therapy" OR "Tranquilizing Drugs" OR BENZODIAZEPINES OR BUSPIRONE OR ALPRAZOLAM OR "ANTIDEPRESSANT DRUGS"

OR "TRICYCLIC ANTIDEPRESSANT DRUGS" OR "Serotonin Reuptake Inhibitors" OR Chlorimipramine OR FLUOXETINE OR PAROXETINE OR SERTRALINE OR CITALOPRAM OR FLUVOXAMINE OR VENLAFAXINE OR Tryptophan OR

"Serotonin Norepinephrine Reuptake Inhibitors" OR LITHIUM OR "ANALGESIC DRUGS" OR Aspirin OR "Adrenergic Blocking Drugs" OR DIURETICS OR "Anti Inflammatory Drugs" OR "Hormone Therapy" OR "Progestational Hormones"

OR PROGESTERONE OR "Oral Contraceptives" OR TESTOSTERONE OR Bromocriptine OR ESTRADIOL OR "Dietary Supplements" OR Vitamins OR CALCIUM OR MAGNESIUM OR ZINC).sh)

***MEDLINE*** *All(R) (Ovid, Advanced Search)*

(conducted 2023-06-13; 849 hits)

((premenstruum OR "premenstrual syndrome" OR "premenstrual symptom*" OR PMS OR "premenstrual dysphoric disorder*" OR PMDD OR "premenstrual disturbance*" OR "premenstrual disorder*" OR PMD

OR "premenstrual complaint*" OR "premenstrual ailment" OR "premenstrual burden" OR "premenstrual tension" OR PMT OR "premenstrual tension syndrome" OR PMTS OR "late luteal

phase dysphoric disorder" OR LLPDD OR "premenstrual dysphoria" OR "premenstrual distress" OR "premenstrual mood change*" OR "premenstrual depression" OR premenstrual).ti,ab,kw

OR ("Premenstrual Syndrome" or "Premenstrual Dysphoric Disorder").sh)

AND

(("controlled stud*" OR "controlled trial*" OR "clinical trial*" OR randomi?ed OR "randomi?ed controlled trial*" OR RCT OR blind OR "double blind").ti,ab,kw

OR ("Double-Blind Method" or "Randomized Controlled Trial" or "Clinical Trial").sh)

AND

((phytopharmaceutical* OR "herbal medicine" OR "complementary medicine" OR "alternative medicine" OR "st. john's wort" OR "saint john's wort" OR "hypericum perforatum" OR "vitex agnus castus" OR

"black cohosh" OR "actaea racemosa" OR "neptune krill" OR valerian OR "valeriana officinalis" OR balm OR melissa OR saffron OR crocus OR "crocus sativus" OR "evening primrose oil" OR

pharmacotherapy OR pharmacotherapeutics OR "pharmacological treatment*" OR "non-hormonal treatment*" OR anxiolytic* OR tranquilizer* OR benzodiazepine OR buspirone OR alprazolam OR antidepressant* OR

"tricyclic antidepressant*" OR "serotonin reuptake inhibitor*" OR SRI OR "selective serotonin reuptake inhibitor*" OR SSRI OR clomipramine OR fluoxetine OR paroxetine OR sertraline OR

citalopram OR escitalopram OR fluvoxamine OR venlafaxine OR "L-tryptophan" OR "norepinephrine reuptake inhibitor*" OR NRI OR "noradrenaline reuptake inhibitor*" OR NERI OR

"adrenergic reuptake inhibitor*" OR ARI OR Lithium OR analgesic* OR paracetamol OR acetaminophen OR aspirin OR "acetylsalicylic acid" OR ASA OR "beta-blocker*" OR diuretics OR spironolactone OR

"nonsteroidal anti-inflammatory drug*" OR NSAID OR "mefenamic acid" OR ibuprofen OR "hormonal treatment*" OR "hormone therap*" OR progestins OR progesterone OR drospirenone OR norgestimate OR

levonorgestrel OR desogestrel OR norethindrone OR "cyproterone acetate" OR "oral contraceptive*" OR "combined oral contraceptive pill" OR COCP OR "gonadotropin-releasing hormone agonist" OR

"GnRH agonist*" OR "GnRH agonist analogs" OR progestogens OR norethisterone OR dydrogesterone OR "medroxyprogesterone acetate" OR "depo-provera" OR testosterone OR bromocriptine OR mifepristone

OR "RU-486" OR "cyproterone acetate" OR tibolone OR danazol OR gestrinone OR estradiol OR oestradiol OR "dietary supplement*" OR "vitamin B6" OR "vitamin D" OR calcium OR magnesium OR zinc).ti,ab,kw

OR ("Herbal Medicine" OR "Complementary Therapies" OR Hypericum OR Vitex OR Cimicifuga OR Valerian OR Melissa OR Crocus OR "Oenothera biennis" OR "Drug Therapy" OR "Anti-Anxiety Agents" OR "Tranquilizing Agents"

OR Benzodiazepines OR Buspirone OR Alprazolam OR "Antidepressive Agents" OR "Antidepressive Agents, Tricyclic" OR "Serotonin and Noradrenaline Reuptake Inhibitors" OR "Serotonin Uptake Inhibitors"

OR Clomipramine OR Fluoxetine OR Paroxetine OR Sertraline OR Citalopram OR Fluvoxamine OR "Venlafaxine Hydrochloride" OR Tryptophan OR "Adrenergic Uptake Inhibitors" OR "Lithium" OR Analgesics OR Acetaminophen

OR Acetaminophen OR Aspirin OR "Adrenergic beta-3 Receptor Antagonists" OR Diuretics OR Spironolactone OR "Anti-Inflammatory Agents, Non-Steroidal" OR "Mefenamic Acid" OR Ibuprofen OR Progestins

OR Progesterone OR Levonorgestrel OR Desogestrel OR Norethindrone OR "Cyproterone Acetate" OR "Contraceptives, Oral" OR "Contraceptives, Oral, Combined" OR Progestins OR Norethindrone OR Dydrogesterone

OR "Medroxyprogesterone Acetate" OR Testosterone OR Bromocriptine OR Mifepristone OR "Cyproterone Acetate" OR Danazol OR Gestrinone OR Estradiol OR "Dietary Supplements" OR "Vitamin B 6" OR "Vitamin D" OR

Calcium OR Magnesium OR Zinc).sh)

***PubMed***

(conducted 2023-06-13; 804 hits)

((premenstruum[Title/Abstract] OR "premenstrual syndrome"[Title/Abstract] OR "premenstrual symptoms"[Title/Abstract] OR PMS[Title/Abstract] OR "premenstrual dysphoric disorder"[Title/Abstract] OR PMDD[Title/Abstract] OR

"premenstrual disturbance"[Title/Abstract] OR "premenstrual disorder"[Title/Abstract] OR PMD[Title/Abstract] OR "premenstrual complaints"[Title/Abstract] OR "premenstrual tension"[Title/Abstract] OR PMT[Title/Abstract]

OR "premenstrual tension syndrome"[Title/Abstract] OR PMTS[Title/Abstract] OR "late luteal phase dysphoric disorder"[Title/Abstract] OR LLPDD[Title/Abstract] OR "premenstrual dysphoria"[Title/Abstract] OR

"premenstrual distress"[Title/Abstract] OR "premenstrual mood changes" OR "premenstrual depression"[Title/Abstract] OR premenstrual[Title/Abstract])

OR ("Premenstrual Syndrome"[MeSH Terms] or "Premenstrual Dysphoric Disorder"[MeSH Terms]))

AND

(("controlled study"[Title/Abstract] OR "controlled trial"[Title/Abstract] OR "clinical trial"[Title/Abstract] OR randomized[Title/Abstract] OR randomised[Title/Abstract] OR "randomized controlled trial"[Title/Abstract]

OR "randomised controlled trial"[Title/Abstract] OR RCT[Title/Abstract] OR blind[Title/Abstract] OR "double blind"[Title/Abstract])

OR ("Double-Blind Method"[MeSH Terms] or "Randomized Controlled Trials as Topic"[MeSH Terms] or "Clinical Trials as Topic"[MeSH Terms]))

AND

((phytopharmaceutical[Title/Abstract] OR "herbal medicine"[Title/Abstract] OR "complementary medicine"[Title/Abstract] OR "alternative medicine"[Title/Abstract] OR "st. john's wort"[Title/Abstract]

OR "saint john's wort"[Title/Abstract] OR "hypericum perforatum"[Title/Abstract] OR "vitex agnus castus"[Title/Abstract] OR "black cohosh"[Title/Abstract] OR "actaea racemosa"[Title/Abstract] OR

valerian[Title/Abstract] OR "valeriana officinalis"[Title/Abstract] OR balm[Title/Abstract] OR melissa[Title/Abstract] OR saffron[Title/Abstract] OR crocus[Title/Abstract]

OR "crocus sativus"[Title/Abstract] OR "evening primrose oil"[Title/Abstract] OR pharmacotherapy[Title/Abstract] OR pharmacotherapeutics[Title/Abstract] OR "pharmacological treatment"[Title/Abstract]

OR "non-hormonal treatment"[Title/Abstract] OR anxiolytics[Title/Abstract] OR tranquilizer[Title/Abstract] OR benzodiazepine[Title/Abstract] OR buspirone[Title/Abstract] OR alprazolam[Title/Abstract]

OR antidepressant[Title/Abstract] OR "tricyclic antidepressant"[Title/Abstract] OR "serotonin reuptake inhibitor"[Title/Abstract] OR SRI[Title/Abstract] OR "selective serotonin reuptake inhibitor"[Title/Abstract]

OR SSRI[Title/Abstract] OR clomipramine[Title/Abstract] OR fluoxetine[Title/Abstract] OR paroxetine[Title/Abstract] OR sertraline[Title/Abstract] OR citalopram[Title/Abstract] OR escitalopram[Title/Abstract]

OR fluvoxamine[Title/Abstract] OR venlafaxine[Title/Abstract] OR "L-tryptophan"[Title/Abstract] OR "norepinephrine reuptake inhibitor"[Title/Abstract] OR NRI[Title/Abstract] OR

"noradrenaline reuptake inhibitor"[Title/Abstract] OR NERI[Title/Abstract] OR ARI[Title/Abstract] OR Lithium[Title/Abstract] OR analgesics[Title/Abstract]

OR paracetamol[Title/Abstract] OR acetaminophen[Title/Abstract] OR aspirin[Title/Abstract] OR "acetylsalicylic acid"[Title/Abstract] OR ASA[Title/Abstract] OR "beta-blocker"[Title/Abstract] OR diuretics[Title/Abstract]

OR spironolactone[Title/Abstract] OR "nonsteroidal anti-inflammatory drug"[Title/Abstract] OR NSAID[Title/Abstract] OR "mefenamic acid"[Title/Abstract] OR ibuprofen[Title/Abstract] OR "hormonal treatment"[Title/Abstract]

OR "hormone therapy"[Title/Abstract] OR progestins[Title/Abstract] OR progesterone[Title/Abstract] OR drospirenone[Title/Abstract] OR norgestimate[Title/Abstract] OR levonorgestrel[Title/Abstract]

OR desogestrel[Title/Abstract] OR norethindrone[Title/Abstract] OR "cyproterone acetate"[Title/Abstract] OR "oral contraceptive"[Title/Abstract] OR "combined oral contraceptive pill"[Title/Abstract] OR COCP[Title/Abstract]

OR "gonadotropin-releasing hormone agonist"[Title/Abstract] OR "GnRH agonist"[Title/Abstract] OR "GnRH agonist analogs"[Title/Abstract] OR progestogens[Title/Abstract] OR norethisterone[Title/Abstract]

OR dydrogesterone[Title/Abstract] OR "medroxyprogesterone acetate"[Title/Abstract] OR "depo-provera"[Title/Abstract] OR testosterone[Title/Abstract] OR bromocriptine[Title/Abstract] OR mifepristone[Title/Abstract]

OR "RU-486"[Title/Abstract] OR "cyproterone acetate"[Title/Abstract] OR tibolone[Title/Abstract] OR danazol[Title/Abstract] OR gestrinone[Title/Abstract] OR estradiol[Title/Abstract] OR oestradiol[Title/Abstract]

OR "dietary supplement"[Title/Abstract] OR "dietary supplements"[Title/Abstract] OR "vitamin B6"[Title/Abstract] OR "vitamin D"[Title/Abstract] OR calcium[Title/Abstract] OR magnesium[Title/Abstract] OR zinc[Title/Abstract])

OR

("Herbal Medicine"[MeSH Terms] OR "Complementary Therapies"[MeSH Terms] OR Hypericum[MeSH Terms] OR Vitex[MeSH Terms] OR Cimicifuga[MeSH Terms] OR Valerian[MeSH Terms] OR Melissa[MeSH Terms] OR Crocus[MeSH Terms]

OR "Oenothera biennis"[MeSH Terms] OR "Drug Therapy"[MeSH Terms] OR "Anti-Anxiety Agents"[MeSH Terms] OR "Tranquilizing Agents"[MeSH Terms] OR Benzodiazepines[MeSH Terms] OR Buspirone[MeSH Terms] OR Alprazolam[MeSH Terms]

OR "Antidepressive Agents"[MeSH Terms] OR "Antidepressive Agents, Tricyclic"[MeSH Terms] OR Serotonin[MeSH Terms] OR "Serotonin Uptake Inhibitors"[MeSH Terms]

OR Clomipramine[MeSH Terms] OR Fluoxetine[MeSH Terms] OR Paroxetine[MeSH Terms] OR Sertraline[MeSH Terms] OR Citalopram[MeSH Terms] OR Fluvoxamine[MeSH Terms] OR "Venlafaxine Hydrochloride"[MeSH Terms]

OR Tryptophan[MeSH Terms] OR "Adrenergic Uptake Inhibitors"[MeSH Terms] OR "Lithium"[MeSH Terms] OR Analgesics[MeSH Terms] OR Acetaminophen[MeSH Terms] OR Acetaminophen[MeSH Terms] OR Aspirin[MeSH Terms]

OR "Adrenergic beta-3 Receptor Antagonists"[MeSH Terms] OR Diuretics[MeSH Terms] OR Spironolactone[MeSH Terms] OR "Anti-Inflammatory Agents, Non-Steroidal"[MeSH Terms] OR "Mefenamic Acid"[MeSH Terms]

OR Ibuprofen[MeSH Terms] OR Progestins[MeSH Terms] OR Progesterone[MeSH Terms] OR Levonorgestrel[MeSH Terms] OR Desogestrel[MeSH Terms] OR Norethindrone[MeSH Terms] OR "Cyproterone Acetate"[MeSH Terms]

OR "Contraceptives, Oral"[MeSH Terms] OR "Contraceptives, Oral, Combined"[MeSH Terms] OR Progestins[MeSH Terms] OR Norethindrone[MeSH Terms] OR Dydrogesterone[MeSH Terms]

OR "Medroxyprogesterone Acetate"[MeSH Terms] OR Testosterone[MeSH Terms] OR Bromocriptine[MeSH Terms] OR Mifepristone[MeSH Terms] OR "Cyproterone Acetate"[MeSH Terms] OR Danazol[MeSH Terms] OR Gestrinone[MeSH Terms]

OR Estradiol[MeSH Terms] OR "Dietary Supplements"[MeSH Terms] OR "Vitamin B 6"[MeSH Terms] OR "Vitamin D"[MeSH Terms] OR Calcium[MeSH Terms] OR Magnesium[MeSH Terms] OR Zinc[MeSH Terms]))

***EMBASE*** *(Ovid, Advanced Search)*

(conducted 2023-06-13; 11044 hits)

((premenstruum OR "premenstrual syndrome" OR "premenstrual symptom*" OR PMS OR "premenstrual dysphoric disorder*" OR PMDD OR "premenstrual disturbance*" OR "premenstrual disorder*" OR PMD

OR "premenstrual complaint*" OR "premenstrual ailment" OR "premenstrual burden" OR "premenstrual tension" OR PMT OR "premenstrual tension syndrome" OR PMTS OR "late luteal

phase dysphoric disorder" OR LLPDD OR "premenstrual dysphoria" OR "premenstrual distress" OR "premenstrual mood change*" OR "premenstrual depression" OR premenstrual).ti,ab,kw

OR ("premenstrual syndrome" OR "premenstrual dysphoric disorder" OR "menstrual cycle").sh)

AND

(("controlled stud*" OR "controlled trial*" OR "clinical trial*" OR randomi?ed OR "randomi?ed controlled trial*" OR RCT OR blind OR "double blind").ti,ab,kw

OR ("controlled study" OR "clinical trial" OR "double blind procedure" OR "randomized controlled trial").sh)

AND

((phytopharmaceutical* OR "herbal medicine" OR "complementary medicine" OR "alternative medicine" OR "st. john's wort" OR "saint john's wort" OR "hypericum perforatum" OR "vitex agnus castus" OR

"black cohosh" OR "actaea racemosa" OR "neptune krill" OR valerian OR "valeriana officinalis" OR balm OR melissa OR saffron OR crocus OR "crocus sativus" OR "evening primrose oil" OR

pharmacotherapy OR pharmacotherapeutics OR "pharmacological treatment*" OR "non-hormonal treatment*" OR anxiolytic* OR tranquilizer* OR benzodiazepine OR buspirone OR alprazolam OR antidepressant* OR

"tricyclic antidepressant*" OR "serotonin reuptake inhibitor*" OR SRI OR "selective serotonin reuptake inhibitor*" OR SSRI OR clomipramine OR fluoxetine OR paroxetine OR sertraline OR

citalopram OR escitalopram OR fluvoxamine OR venlafaxine OR "L-tryptophan" OR "norepinephrine reuptake inhibitor*" OR NRI OR "noradrenaline reuptake inhibitor*" OR NERI OR

"adrenergic reuptake inhibitor*" OR ARI OR Lithium OR analgesic* OR paracetamol OR acetaminophen OR aspirin OR "acetylsalicylic acid" OR ASA OR "beta-blocker*" OR diuretics OR spironolactone OR

"nonsteroidal anti-inflammatory drug*" OR NSAID OR "mefenamic acid" OR ibuprofen OR "hormonal treatment*" OR "hormone therap*" OR progestins OR progesterone OR drospirenone OR norgestimate OR

levonorgestrel OR desogestrel OR norethindrone OR "cyproterone acetate" OR "oral contraceptive*" OR "combined oral contraceptive pill" OR COCP OR "gonadotropin-releasing hormone agonist" OR

"GnRH agonist*" OR "GnRH agonist analogs" OR progestogens OR norethisterone OR dydrogesterone OR "medroxyprogesterone acetate" OR "depo-provera" OR testosterone OR bromocriptine OR mifepristone

OR "RU-486" OR "cyproterone acetate" OR tibolone OR danazol OR gestrinone OR estradiol OR oestradiol OR "dietary supplement*" OR "vitamin B6" OR "vitamin D" OR calcium OR magnesium OR zinc).ti,ab,kw

OR (phytochemical OR "herbal medicine" OR "alternative medicine" OR "Hypericum perforatum" OR "vitex agnus castus" OR "Actaea racemosa" OR valerian OR "valeriana officinalis" OR "Melissa officinalis" OR

"Crocus sativus" OR "primrose oil" OR "drug therapy" OR "anxiolytic agent" OR "tranquilizer" OR "benzodiazepine" OR buspirone OR alprazolam OR "antidepressant agent" OR "tricyclic antidepressant agent"

OR "serotonin uptake inhibitor" OR clomipramine OR fluoxetine OR paroxetine OR sertraline OR citalopram OR escitalopram OR fluvoxamine OR venlafaxine OR tryptophan OR "noradrenalin uptake inhibitor" OR

lithium OR "analgesic agent" OR paracetamol OR "acetylsalicylic acid" OR "beta adrenergic receptor blocking agent" OR "diuretic agent" OR spironolactone OR "nonsteroid antiinflammatory agent" OR

"mefenamic acid" OR ibuprofen OR "hormonal therapy" OR gestagen OR progesterone OR drospirenone OR norgestimate OR levonorgestrel OR desogestrel OR norethisterone OR "cyproterone acetate" OR

"oral contraceptive agent" OR "oral contraception" OR "gonadorelin agonist" OR norethisterone OR dydrogesterone OR "medroxyprogesterone acetate" OR testosterone OR bromocriptine OR mifepristone OR

"cyproterone acetate" OR tibolone OR danazol OR gestrinone OR estradiol OR "dietary supplement" OR pyridoxine OR "vitamin D" OR calcium OR magnesium OR zinc).sh)

***SCOPUS*** *(Advanced Search)*

(conducted 2023-06-13; 2469 hits)

( TITLE-ABS-KEY ( premenstruum OR "premenstrual syndrome" OR "premenstrual symptom*" OR pms OR "premenstrual dysphoric disorder*" OR pmdd OR "premenstrual disturbance*" OR "premenstrual disorder*"

OR pmd OR "premenstrual complaint*" OR "premenstrual ailment" OR "premenstrual burden" OR "premenstrual tension" OR pmt OR "premenstrual tension syndrome" OR pmts OR "late luteal phase dysphoric disorder"

OR llpdd OR "premenstrual dysphoria" OR "premenstrual distress" OR "premenstrual mood change*" OR "premenstrual depression" OR premenstrual ) ) AND ( TITLE-ABS-KEY ( "controlled stud*" OR "controlled trial*"

OR "clinical trial*" OR randomi?ed OR "randomi?ed controlled trial*" OR rct OR blind OR "double blind" ) AND NOT PM ) AND ( TITLE-ABS-KEY ( phytopharmaceutical* OR "herbal medicine" OR "complementary medicine"

OR "alternative medicine" OR "st. john's wort" OR "saint john's wort" OR "hypericum perforatum" OR "vitex agnus castus" OR "black cohosh" OR "actaea racemosa" OR "neptune krill" OR valerian

OR "valeriana officinalis" OR balm OR melissa OR saffron OR crocus OR "crocus sativus" OR "evening primrose oil" OR pharmacotherapy OR pharmacotherapeutics OR "pharmacological treatment*"

OR "non-hormonal treatment*" OR anxiolytic* OR tranquilizer* OR benzodiazepine OR buspirone OR alprazolam OR antidepressant* OR "tricyclic antidepressant*" OR "serotonin reuptake inhibitor*"

OR sri OR "selective serotonin reuptake inhibitor*" OR ssri OR clomipramine OR fluoxetine OR paroxetine OR sertraline OR citalopram OR escitalopram OR fluvoxamine OR venlafaxine OR "L-tryptophan"

OR "norepinephrine reuptake inhibitor*" OR nri OR "noradrenaline reuptake inhibitor*" OR neri OR "adrenergic reuptake inhibitor*" OR ari OR lithium OR analgesic* OR paracetamol OR acetaminophen

OR aspirin OR "acetylsalicylic acid" OR asa OR "beta-blocker*" OR diuretics OR spironolactone OR "nonsteroidal anti-inflammatory drug*" OR nsaid OR "mefenamic acid" OR ibuprofen OR "hormonal treatment*"

OR "hormone therap*" OR progestins OR progesterone OR drospirenone OR norgestimate OR levonorgestrel OR desogestrel OR norethindrone OR "cyproterone acetate" OR "oral contraceptive*"

OR "combined oral contraceptive pill" OR cocp OR "gonadotropin-releasing hormone agonist" OR "GnRH agonist*" OR "GnRH agonist analogs" OR progestogens OR norethisterone OR dydrogesterone

OR "medroxyprogesterone acetate" OR "depo-provera" OR testosterone OR bromocriptine OR mifepristone OR "RU-486" OR "cyproterone acetate" OR tibolone OR danazol OR gestrinone OR estradiol OR oestradiol

OR "dietary supplement*" OR "vitamin B6" OR "vitamin D" OR calcium OR magnesium OR zinc ) )

***Web of Science*** *(All Databases, Topic)*

(conducted 2023-06-13; 1419 hits)

((premenstruum OR "premenstrual syndrome" OR "premenstrual symptom*" OR PMS OR "premenstrual dysphoric disorder*" OR PMDD OR "premenstrual disturbance*" OR "premenstrual disorder*" OR PMD OR "premenstrual complaint*"

OR "premenstrual ailment" OR "premenstrual burden" OR "premenstrual tension" OR PMT OR "premenstrual tension syndrome" OR PMTS OR "late luteal phase dysphoric disorder" OR LLPDD OR "premenstrual dysphoria"

OR "premenstrual distress" OR "premenstrual mood change*" OR "premenstrual depression" OR premenstrual)

AND

("controlled stud*" OR "controlled trial*" OR "clinical trial*" OR randomi?ed OR "randomi?ed controlled trial*" OR RCT OR blind OR "double blind")

AND

(phytopharmaceutical* OR "herbal medicine" OR "complementary medicine" OR "alternative medicine" OR "st. john's wort" OR "saint john's wort" OR "hypericum perforatum" OR "vitex agnus castus" OR "black cohosh"

OR "actaea racemosa" OR "neptune krill" OR valerian OR "valeriana officinalis" OR balm OR melissa OR saffron OR crocus OR "crocus sativus" OR "evening primrose oil" OR pharmacotherapy OR pharmacotherapeutics

OR "pharmacological treatment*" OR "non-hormonal treatment*" OR anxiolytic* OR tranquilizer* OR benzodiazepine OR buspirone OR alprazolam OR antidepressant* OR "tricyclic antidepressant*" OR "serotonin reuptake inhibitor*"

OR SRI OR "selective serotonin reuptake inhibitor*" OR SSRI OR clomipramine OR fluoxetine OR paroxetine OR sertraline OR citalopram OR escitalopram OR fluvoxamine OR venlafaxine OR "L-tryptophan"

OR "norepinephrine reuptake inhibitor*" OR NRI OR "noradrenaline reuptake inhibitor*" OR NERI OR "adrenergic reuptake inhibitor*" OR ARI OR Lithium OR analgesic* OR paracetamol OR acetaminophen OR aspirin

OR "acetylsalicylic acid" OR ASA OR "beta-blocker*" OR diuretics OR spironolactone OR "nonsteroidal anti-inflammatory drug*" OR NSAID OR "mefenamic acid" OR ibuprofen OR "hormonal treatment*" OR "hormone therap*"

OR progestins OR progesterone OR drospirenone OR norgestimate OR levonorgestrel OR desogestrel OR norethindrone OR "cyproterone acetate" OR "oral contraceptive*" OR "combined oral contraceptive pill" OR COCP

OR "gonadotropin-releasing hormone agonist" OR "GnRH agonist*" OR "GnRH agonist analogs" OR progestogens OR norethisterone OR dydrogesterone OR "medroxyprogesterone acetate" OR "depo-provera" OR testosterone

OR bromocriptine OR mifepristone OR "RU-486" OR "cyproterone acetate" OR tibolone OR danazol OR gestrinone OR estradiol OR oestradiol OR "dietary supplement*" OR "vitamin B6" OR "vitamin D" OR calcium OR magnesium OR zinc))

***CINAHL (EBSCO****, Advanced Search)*

(conducted 2023-06-13; 441 hits)

(TI (premenstruum OR "premenstrual syndrome" OR "premenstrual symptom*" OR PMS OR "premenstrual dysphoric disorder*" OR PMDD OR "premenstrual disturbance*" OR "premenstrual disorder*" OR PMD OR "premenstrual complaint*"

OR "premenstrual ailment" OR "premenstrual burden" OR "premenstrual tension" OR PMT OR "premenstrual tension syndrome" OR PMTS OR "late luteal

phase dysphoric disorder" OR LLPDD OR "premenstrual dysphoria" OR "premenstrual distress" OR "premenstrual mood change*" OR "premenstrual depression" OR premenstrual) OR AB (premenstruum OR "premenstrual syndrome"

OR "premenstrual symptom*" OR PMS OR "premenstrual dysphoric disorder*" OR PMDD OR "premenstrual disturbance*" OR "premenstrual disorder*" OR PMD OR "premenstrual complaint*"

OR "premenstrual ailment" OR "premenstrual burden" OR "premenstrual tension" OR PMT OR "premenstrual tension syndrome" OR PMTS OR "late luteal

phase dysphoric disorder" OR LLPDD OR "premenstrual dysphoria" OR "premenstrual distress" OR "premenstrual mood change*" OR "premenstrual depression" OR premenstrual) OR SU ("Premenstrual Syndrome" OR

"Premenstrual Dysphoric Disorder" OR "Luteal Phase"))

AND

(TI ("controlled stud*" OR "controlled trial*" OR "clinical trial*" OR randomi?ed OR "randomi?ed controlled trial*" OR RCT OR blind OR "double blind") OR AB ("controlled stud*" OR "controlled trial*" OR "clinical trial*"

OR randomi?ed OR "randomi?ed controlled trial*" OR RCT OR blind OR "double blind") OR SU ("Case Control Studies" OR "Clinical Trials" OR "Randomized Controlled Trials" OR "Single-Blind Studies" OR "Double-Blind Studies"

OR "Triple-Blind Studies"))

AND

(TI (phytopharmaceutical* OR "herbal medicine" OR "complementary medicine" OR "alternative medicine" OR "st. john's wort" OR "saint john's wort" OR "hypericum perforatum" OR "vitex agnus castus" OR "black cohosh"

OR "actaea racemosa" OR "neptune krill" OR valerian OR "valeriana officinalis" OR balm OR melissa OR saffron OR crocus OR "crocus sativus" OR "evening primrose oil" OR pharmacotherapy OR pharmacotherapeutics

OR "pharmacological treatment*" OR "non-hormonal treatment*" OR anxiolytic* OR tranquilizer* OR benzodiazepine OR buspirone OR alprazolam OR antidepressant* OR "tricyclic antidepressant*" OR "serotonin reuptake inhibitor*"

OR SRI OR "selective serotonin reuptake inhibitor*" OR SSRI OR clomipramine OR fluoxetine OR paroxetine OR sertraline OR citalopram OR escitalopram OR fluvoxamine OR venlafaxine OR "L-tryptophan"

OR "norepinephrine reuptake inhibitor*" OR NRI OR "noradrenaline reuptake inhibitor*" OR NERI OR "adrenergic reuptake inhibitor*" OR ARI OR Lithium OR analgesic* OR paracetamol OR acetaminophen OR aspirin

OR "acetylsalicylic acid" OR ASA OR "beta-blocker*" OR diuretics OR spironolactone OR "nonsteroidal anti-inflammatory drug*" OR NSAID OR "mefenamic acid" OR ibuprofen OR "hormonal treatment*" OR "hormone therap*"

OR progestins OR progesterone OR drospirenone OR norgestimate OR levonorgestrel OR desogestrel OR norethindrone OR "cyproterone acetate" OR "oral contraceptive*" OR "combined oral contraceptive pill" OR COCP

OR "gonadotropin-releasing hormone agonist" OR "GnRH agonist*" OR "GnRH agonist analogs" OR progestogens OR norethisterone OR dydrogesterone OR "medroxyprogesterone acetate" OR "depo-provera" OR testosterone

OR bromocriptine OR mifepristone OR "RU-486" OR "cyproterone acetate" OR tibolone OR danazol OR gestrinone OR estradiol OR oestradiol OR "dietary supplement*" OR "vitamin B6" OR "vitamin D" OR calcium OR magnesium OR zinc)

OR AB (phytopharmaceutical* OR "herbal medicine" OR "complementary medicine" OR "alternative medicine" OR "st. john's wort" OR "saint john's wort" OR "hypericum perforatum" OR "vitex agnus castus" OR "black cohosh"

OR "actaea racemosa" OR "neptune krill" OR valerian OR "valeriana officinalis" OR balm OR melissa OR saffron OR crocus OR "crocus sativus" OR "evening primrose oil" OR pharmacotherapy OR pharmacotherapeutics

OR "pharmacological treatment*" OR "non-hormonal treatment*" OR anxiolytic* OR tranquilizer* OR benzodiazepine OR buspirone OR alprazolam OR antidepressant* OR "tricyclic antidepressant*" OR "serotonin reuptake inhibitor*"

OR SRI OR "selective serotonin reuptake inhibitor*" OR SSRI OR clomipramine OR fluoxetine OR paroxetine OR sertraline OR citalopram OR escitalopram OR fluvoxamine OR venlafaxine OR "L-tryptophan"

OR "norepinephrine reuptake inhibitor*" OR NRI OR "noradrenaline reuptake inhibitor*" OR NERI OR "adrenergic reuptake inhibitor*" OR ARI OR Lithium OR analgesic* OR paracetamol OR acetaminophen OR aspirin

OR "acetylsalicylic acid" OR ASA OR "beta-blocker*" OR diuretics OR spironolactone OR "nonsteroidal anti-inflammatory drug*" OR NSAID OR "mefenamic acid" OR ibuprofen OR "hormonal treatment*" OR "hormone therap*"

OR progestins OR progesterone OR drospirenone OR norgestimate OR levonorgestrel OR desogestrel OR norethindrone OR "cyproterone acetate" OR "oral contraceptive*" OR "combined oral contraceptive pill" OR COCP

OR "gonadotropin-releasing hormone agonist" OR "GnRH agonist*" OR "GnRH agonist analogs" OR progestogens OR norethisterone OR dydrogesterone OR "medroxyprogesterone acetate" OR "depo-provera" OR testosterone

OR bromocriptine OR mifepristone OR "RU-486" OR "cyproterone acetate" OR tibolone OR danazol OR gestrinone OR estradiol OR oestradiol OR "dietary supplement*" OR "vitamin B6" OR "vitamin D" OR calcium OR magnesium OR zinc)

OR SU ("Medicine, Herbal" OR "Alternative Therapies" OR "St. John's Wort" OR "Black Cohosh" OR Valerian OR "Lemon Balm" OR "Evening Primrose" OR "Drug Therapy" OR "Pharmacological and Biological Treatments" OR

"Antianxiety Agents" OR "Tranquilizing Agents" OR "Antianxiety Agents, Benzodiazepine" OR Buspirone OR Alprazolam OR "Antidepressive Agents" OR "Antidepressive Agents, Tricyclic" OR "Serotonin Uptake Inhibitors"

OR Clomipramine OR Fluoxetine OR Paroxetine OR "Sertraline Hydrochloride" OR Citalopram OR "Fluvoxamine Maleate" OR Venlafaxine OR Tryptophan OR "Adrenergic Uptake Inhibitors" OR Lithium OR Analgesics OR Acetaminophen

OR Aspirin OR "Adrenergic Beta-Antagonists" OR Diuretics OR Spironolactone OR "Antiinflammatory Agents, Non-Steroidal" OR Ibuprofen OR "Progestational Hormones" OR Progesterone OR Levonorgestrel OR

"Contraceptives, Oral" OR "Contraceptives, Oral Combined" OR Gonadorelin OR "Medroxyprogesterone Acetate" OR Testosterone OR Bromocriptine OR Mifepristone OR Danazol OR Estradiol OR "Dietary Supplements"

OR Pyridoxine OR "Vitamin D" OR Calcium OR Magnesium OR Zinc))

***COCHRANE*** *Library (Advanced Search, Search Manager)*

(conducted 2023-06-13; 852 hits (68 Reviews, 13 Protocols, 768 Trials, 3 Clinical Answers))

(premenstruum OR "premenstrual syndrome" OR "premenstrual symptom*" OR PMS OR "premenstrual dysphoric disorder*" OR PMDD OR "premenstrual disturbance*" OR "premenstrual disorder*" OR PMD OR "premenstrual complaint*"

OR "premenstrual ailment" OR "premenstrual burden" OR "premenstrual tension" OR PMT OR "premenstrual tension syndrome" OR PMTS OR "late luteal phase dysphoric disorder" OR LLPDD OR "premenstrual dysphoria"

OR "premenstrual distress" OR "premenstrual mood change*" OR "premenstrual depression" OR premenstrual:ti,ab,kw) AND ("controlled stud*" OR "controlled trial*" OR "clinical trial*" OR randomi?ed OR "randomi?ed controlled trial*"

OR RCT OR blind OR "double blind":ti,ab,kw) AND (phytopharmaceutical* OR "herbal medicine" OR "complementary medicine" OR "alternative medicine" OR "st. john's wort" OR "saint john's wort" OR "hypericum perforatum" OR "vitex agnus castus" OR "black cohosh"

OR "actaea racemosa" OR "neptune krill" OR valerian OR "valeriana officinalis" OR balm OR melissa OR saffron OR crocus OR "crocus sativus" OR "evening primrose oil" OR pharmacotherapy OR pharmacotherapeutics

OR "pharmacological treatment*" OR "non-hormonal treatment*" OR anxiolytic* OR tranquilizer* OR benzodiazepine OR buspirone OR alprazolam OR antidepressant* OR "tricyclic antidepressant*" OR "serotonin reuptake inhibitor*"

OR SRI OR "selective serotonin reuptake inhibitor*" OR SSRI OR clomipramine OR fluoxetine OR paroxetine OR sertraline OR citalopram OR escitalopram OR fluvoxamine OR venlafaxine OR "L-tryptophan"

OR "norepinephrine reuptake inhibitor*" OR NRI OR "noradrenaline reuptake inhibitor*" OR NERI OR "adrenergic reuptake inhibitor*" OR ARI OR Lithium OR analgesic* OR paracetamol OR acetaminophen OR aspirin

OR "acetylsalicylic acid" OR ASA OR "beta-blocker*" OR diuretics OR spironolactone OR "nonsteroidal anti-inflammatory drug*" OR NSAID OR "mefenamic acid" OR ibuprofen OR "hormonal treatment*" OR "hormone therap*"

OR progestins OR progesterone OR drospirenone OR norgestimate OR levonorgestrel OR desogestrel OR norethindrone OR "cyproterone acetate" OR "oral contraceptive*" OR "combined oral contraceptive pill" OR COCP

OR "gonadotropin-releasing hormone agonist" OR "GnRH agonist*" OR "GnRH agonist analogs" OR progestogens OR norethisterone OR dydrogesterone OR "medroxyprogesterone acetate" OR "depo-provera" OR testosterone

OR bromocriptine OR mifepristone OR "RU-486" OR "cyproterone acetate" OR tibolone OR danazol OR gestrinone OR estradiol OR oestradiol OR "dietary supplement*" OR "vitamin B6" OR "vitamin D" OR calcium OR magnesium OR zinc:ti,ab,kw)
